# Bathurst Burr (*Xanthium spinosum*) Powder—A New Natural Effective Adsorbent for Crystal Violet Dye Removal from Synthetic Wastewaters

**DOI:** 10.3390/ma14195861

**Published:** 2021-10-07

**Authors:** Giannin Mosoarca, Cosmin Vancea, Simona Popa, Sorina Boran

**Affiliations:** Faculty of Industrial Chemistry and Environmental Engineering, Politehnica University Timisoara, Bd. V. Parvan, No. 6, 300223 Timisoara, Romania; giannin.mosoarca@upt.ro (G.M.); sorina.boran@upt.ro (S.B.)

**Keywords:** natural adsorbent, crystal violet, adsorption, isotherm, kinetic, Taguchi method

## Abstract

A new natural adsorbent material, Bathurst burr powder, was used to remove crystal violet dye from synthetic wastewaters. Particle size distribution and SEM and FTIR analyses were performed to characterize it. The effect of the operational adsorption process parameters (pH, ionic strength, initial dye concentration, adsorbent dose, contact time, temperature) onto the adsorption process was evaluated in a batch system. Equilibrium, kinetic, and thermodynamic studies were performed in order to understand the adsorption process. Taguchi method and ANOVA test were used to optimize the dye adsorption conditions and to establish the percentage contribution of each factor, respectively. The accuracy of the Taguchi prediction method was analyzed by correlating the predicted dye removal efficiency with the experimentally determined one. The particle size distribution analysis showed that 82.15% of the adsorbent particles have an average size below 0.5 mm. The adsorption process followed the Langmuir isotherm and pseudo-second order kinetic model. Maximum adsorption capacity value (164.10 mg·g^−1^) was higher compared to many similar adsorbents. The process was endothermic, spontaneous, and favorably involving a physisorption mechanism. The Taguchi method showed that the most influential controllable factor was pH (65% contribution in adsorption efficiency) and the data analysis indicates a very good accuracy of the experimental design (R^2^ = 0.994). The obtained results demonstrated that Bathurst burr powder can be used as a cheap and efficient adsorbent for crystal violet dye removal from aqueous solution.

## 1. Introduction

Crystal violet is a cationic dye used as a textile colorant, paper dye, biological stain, and as veterinary animal drug. It has high toxicity and can cause digestive tract irritation, kidney failure, and cancer [1,2,3,4,5]. Its presence in the aquatic environment, even in low concentrations, leads to plants photosynthesis process disorder. It has high chemical stability, high solubility, and low biodegradability. It is therefore necessary to remove this stain from residual effluents [1,2,3].

Many methods have been used to remove the cationic dyes (inclusive crystal violet) from wastewater and dye solutions. Coagulation, flocculation, electrocoagulation, ozonation and catalytic ozonation, photochemical and photocatalytic degradation, chemical and electrochemical oxidation, membrane processes (ultrafiltration, reverse osmosis), biodegradation, ion exchange, and adsorption have been mentioned in the literature as being effective for this purpose [1,3,4,5,6].

In recent years, the adsorption process has been used more and more often by researchers to achieve the removal of dyes from wastewaters. This is due to the fact that adsorption has many technical and economic advantages such as: high selectivity and efficiency, simplicity and flexibility in operation, good applicability, possibility to reuse the adsorbent, and relatively low costs [4,5,6].

The process becomes extremely cost-effective by using cheap materials that can be found in abundance in nature [7,8,9,10,11]. Several low-cost natural materials have been used for crystal violet dye removal from aqueous solutions: cedar cones [11], *Calotropis procera* leaf [12], *Laminaria japonica* [13], rice bran [13], wheat bran [13], coniferous pinus bark powder [14], sugarcane fiber [15], sawdust [15], coir pith [15], peel of *Cucumis sativa* fruit [16], jackfruit leaf powder [17], pineapple leaf powder [18], papaya seeds [19], breadfruit skin [8], water hyacinth root powder [20], *Eragrostis plana* nees [7], pará chestnut husk [21], date palm leaves powder [22], corn stalk [23], *Ocotea puberula* bark powder [5], *Moringa oleifera pod husk* [24], and almond shells [25].

Bathurst burr (*Xanthium spinosum*) is an herbaceous plant, up to 1 meter tall, with a richly branched stem and full of thorns. It grows especially in hilly and plain areas and also in low mountainous regions. Originally from South America, it has spread widely in Europe, Australia, parts of Africa, Asia, and North America, and is a very invasive plant. It grows on pastures, roadsides, fences, and abandoned lands and has an extraordinary resistance to drought, pollution, and any aggressive environmental factor. It is used in traditional medicine based on its: decongestant, diuretic, anti-inflammatory, disinfectant, diuretic, antidiabetic, and antitumor properties [26,27]. The seeds of the plant contain carboxyatractyloside, which is poisonous to livestock, but animals usually avoid this plant because of its thorns, which can cause them unpleasant wounds [28,29].

The aim of this research study was to remove crystal violet dye from synthetic wastewaters using the Bathurst burr powder as an adsorbent material. The effect of pH, contact time, adsorbent dose, initial dye concentration, temperature, and ionic strength onto the adsorption process was evaluated in the batch system. In order to understand the adsorption mechanism, different isotherms, kinetics, and thermodynamics studies were performed. The Taguchi method was used for adsorption conditions optimization. In addition, the ANOVA analysis was used to assess the result and to determine the contribution percentage of each parameter on the dye removal process.

## 2. Materials and Methods

In order to obtain the adsorbent material, the aerial part of Bathurst burr mature plants were purchased from the company StefMar (Ramnicu Valcea, Romania) whose activity is the processing and packaging of medicinal and aromatic plants. The plants were washed with distilled water and then first dried at room temperature for 3 days and then in an air oven at 105 °C for 24 h. After drying, they were electrically grounded. The obtained powder was passed through a 2 mm sieve. 

SEM analysis and FTIR spectroscopy were performed to characterize the adsorbent material using a Quanta FEG 250 (FEI, Eindhoven, The Netherlands) scanning electron microscope (1600× magnitude) and a Shimadzu Prestige-21 FTIR (Shimadzu, Kyoto, Japan) spectrophotometer. Analyses were compared before and after the dye adsorption process.

In batch adsorption experiments, the influence of various factors affecting the process was studied, at a constant stirring intensity provided by a shaker. The pH was adjusted using 0.1 N NaOH and HCl solutions. The influence of ionic strength was investigated using NaCl as background electrolyte. The crystal violet concentration was measured with a Specord 200 PLUS UV-VIS (Analytik Jena, Jena, Germany) spectrophotometer at a wavelength of 590 nm. Each test was conducted using three independent replicates.

The crystal violet amounts adsorbed at equilibrium (*q_e_*) and the dye removal percentage *R(%)* were calculated with equations (1) and (2) [1,4,7]:

(1) $$q_{e}=\frac{(C_{0}-C_{e})\cdot V}{m},$$

(2) $$R(\%)=\frac{\left(C_{0}-C_{e}\right)}{C_{0}}\cdot 100,$$

where: *C_0_* is the initial crystal violet concentration (mg·L^−1^), *C_e_* is the crystal violet equilibrium concentration (mg·L^−1^), *V* is the solution volume (L), and *m* is the mass of adsorbent (g).

The optimum conditions for crystal violet adsorption were established using Taguchi (L27) experimental design. To achieve this goal, the effect of six factors (at three levels) on dye removal efficiency was followed. These controllable factors and their levels are detailed in Table 1. For the experimental design, the Taguchi method uses an orthogonal matrix (OA) evaluating the signal/noise ratio (S/N) using the “larger is the better” option [30,31,32]. In order to evaluate the results of the Taguchi method and to establish the percentage contribution of each factor to the efficiency of crystal violet removal, an analysis of variance (ANOVA) was realized [30,32]. The necessary calculations were performed with the Minitab 19 Software (version 19.1.1, Minitab LLC, State College, PA, USA).

The desorption study was realized in batch mode, at constant stirring for 2 h, using different desorbing agents (distilled water, 0.1 M HCl, and 0.1 M NaOH).

## 3. Results and Discussion

### 3.1. Adsorbent Characterization

The particle size distribution analysis of the Bathurst burr powder (Supplementary Material, Figure S1) shows that 82.15% of the particles have an average size bellow 0.5 mm and 50% of them are smaller than 0.348 mm.

**Figure 1:**
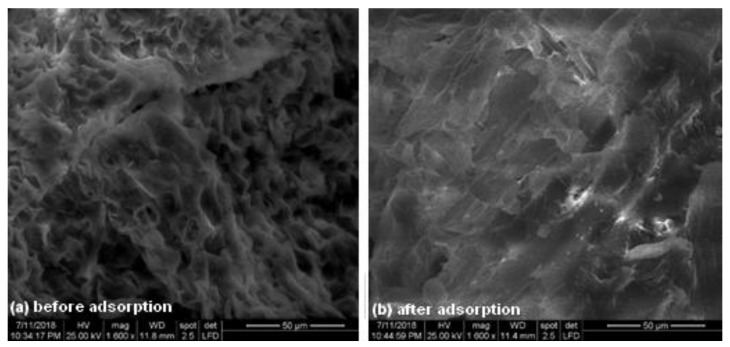
SEM image of adsorbent surface before (**a**) and after (**b**) crystal violet adsorption [adsorption conditions: pH 7; initial dye concentration: 100 mg·L^−1^; contact time: 30 min; adsorbent dose: 2 g·L^−1^; temperature: 294 K, ionic strength: 0 mol L^−1^].

Figure 1 shows the morphology of the adsorbent before and after adsorption. Before adsorption, the surface had many pores that provided a large adsorption surface and a large number of sites available for retaining dye molecules (Figure 1a). After adsorption, the surface of the adsorbent was saturated and covered with crystal violet molecules, which filled the pores and cavities during the process (Figure 1b).

**Figure 2:**
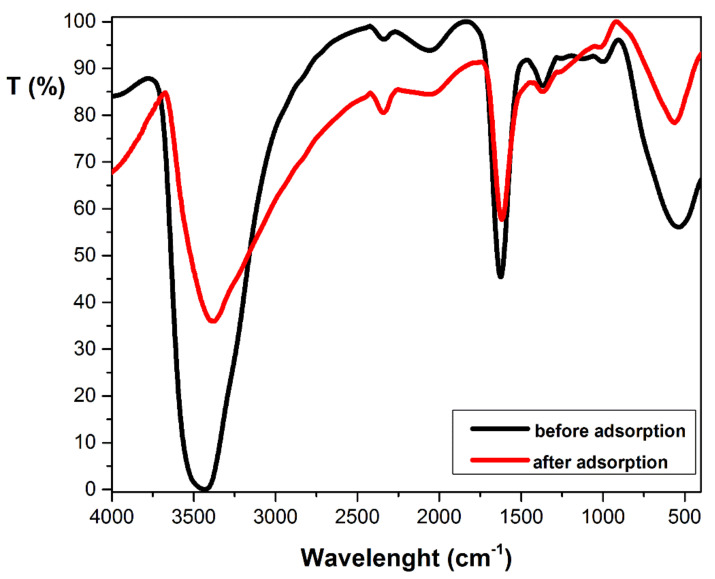
FTIR spectrum of Bathurst burr powder before and after crystal violet adsorption. [adsorption conditions: pH 7; initial dye concentration: 100 mg·L^−1^; contact time: 30 min; adsorbent dose: 2 g·L^−1^; temperature: 294 K, ionic strength: 0 mol L^−1^].

The FTIR spectra of adsorbent before and after adsorption (Figure 2) indicate different bands specific for main functional group existing in cellulose (3448 cm^−1^, 2340 cm^−1^, 1630 cm^−1^, 1368 cm^−1^, and 1000 cm^−1^) [33,34,35,36,37] and hemicellulose (3448 cm^−1^, 1368 cm^−1^) [36,38,39,40]. These assignments are shown in Table 2. After adsorption, the appearance or disappearance of any band was not found. It only presents small changes of the wavenumber, suggesting that the physical interaction or ion exchange can intervene in the adsorption mechanism [41].

### 3.2. Effect of pH and Ionic Strength on Crystal Violet Adsorption

**Figure 3:**
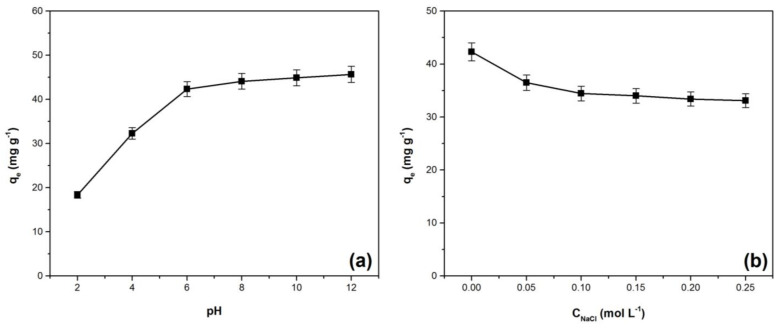
Effect of pH (**a**) and ionic strength (**b**) on adsorption capacity for the crystal violet adsorption onto Bathurst burr powder [adsorption conditions: (**a**): initial dye concentration: 100 mg·L^−1^; contact time: 30 min; adsorbent dose: 2 g·L^−1^; temperature: 294 K, ionic strength: 0 mol L^−1^ (**b**): pH 7, initial dye concentration: 100 mg·L^−1^; contact time: 30 min; adsorbent dose: 2 g·L^−1^; temperature: 294 K].

pH is an important parameter that affects the adsorbent surface charge [42]. The influence of this parameter on the adsorption capacity was followed in the pH range 2–12 (Figure 3a). The adsorption capacity increases with pH, reaching the highest values at basic conditions. A similar phenomenon was observed in other similar studies regarding the adsorption of crystal violet on low-cost adsorbents [8,9,43,44]. The adsorbent materials have different functional groups on the surface. At lower pH these groups will be protonated and the charge on the adsorbent surface will be predominantly positive, therefore, the adsorption process will be more difficult due to electrostatic repulsion with cationic dye molecules. For higher pH values, these functional groups will be deprotonated and the charge on the adsorbent surface will be predominantly negative. This phenomenon favors the adsorption process because there is a strong electrostatic attraction between negatively charged adsorbent surface and the dye cations [18,22,45]. The adsorption capacity on the pH range 8–12 remains practically constant, suggesting that other mechanisms besides electrostatic attraction may be involved in the adsorption process [46,47].

Another important factor that can influence the adsorption process is the ionic strength. The effect of this factor on the adsorption capacity is presented in Figure 3b. The decrease of adsorption capacity as the ionic strength increases is based on the competitive effect between the dye and sodium ions in occupying the available adsorption sites on the adsorbent surface [45,46]. A similar influence of ionic strength on the crystal violet adsorption process has been reported in other previous scientific articles [43,48,49].

### 3.3. Effect of Adsorbent Dose and Initial Dye Concentration on Crystal Violet Adsorption

**Figure 4:**
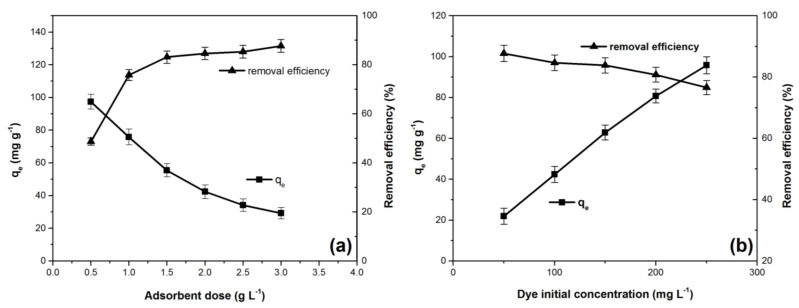
Effect of adsorbent dose (**a**) and initial dye concentration (**b**) on adsorption capacity and removal efficiency for the crystal violet adsorption onto Bathurst burr powder [adsorption conditions: (**a**): pH 7; initial dye concentration: 100 mg·L^−1^; contact time: 30 min; temperature: 294 K, ionic strength: 0 mol L^−1^ (**b**): pH 7, contact time: 30 min; adsorbent dose: 2 g·L^−1^; temperature: 294 K; ionic strength: 0 mol L^−1^].

Figure 4a illustrates the adsorbent dose effect on the crystal violet adsorption process. The increase of adsorbent amount has a positive effect on the dye removal efficiency and a negative effect on the adsorption capacity. A similar phenomenon has been reported for this dye adsorbed using: eggshells [4], *Ocotea puberula* bark powder [5], pinus bark powder [14], lemongrass leaf combined with cellulose acetate [42], functionalized multi-walled carbon nanotubes [50], and inactive biomass of *Diaporthe schini* [51]. The increase of the adsorbent material dose leads to an increase of adsorption surface; therefore, removal efficiency of the dye will be higher [11,14,18,50]. The reported decrease of the adsorption capacity (q_e_) can be explained based on the assumption that even if the adsorption sites number increases, many of them remain unsaturated. Other phenomenon such as agglomeration of adsorbent material particles may also occur [4,23,50,51].

The adsorption capacity increases and the removal efficiency decreases as initial dye concentration increases, as shown in Figure 4b. Similar effects were mentioned for other adsorbents used to retain crystal violet: eggshells [4], *Ananas comosus* (pineapple) leaf powder [18], and corn stalk [23]. The increase of the adsorption capacity can be attributed to the fact that a high initial dye concentration leads to an increase of the concentration gradient between the dye solution and the adsorbent surface, leading to an increase of the driving force that favors the external mass transfer [4,11,18,51,52]. At the same time, the number of collisions between dye molecules and adsorbent particles material are favored, intensifying adsorption [53]. The unfavorable effect of increasing initial dye concentration upon removal efficiency derives from the fact that the active adsorption sites become saturated with the dye molecules accumulated during the process [4,18,50,54].

### 3.4. Equilibrum Study

Adsorption isotherms are used to describe the interactions between adsorbate and adsorbent that take place in the process. The calculated parameters using these isotherms provide information about the properties and affinities of the adsorbent material surface as well as about the adsorption mechanism [1,11,52].

Based on the data presented in Figure 4b (experimental results and working conditions), Langmuir and Freundlich isotherms, in the form of non-linear equations [7,8,43,55,56,57], were used for this purpose:

(3) $$\mathrm{Langmuir}\mathrm{isotherm}\mathrm{non}-\mathrm{linear}\mathrm{equation}:q_{e}=\frac{q_{m}\cdot K_{L}\cdot C_{e}}{1+K_{L}\cdot C_{e}},$$

(4) $$\mathrm{Freundlich}\mathrm{isotherm}\mathrm{non}-\mathrm{linear}\mathrm{equation}:q_{e}=K_{F}\cdot C_{e}^{1/n_{F}},$$

where: *q_m_* is the maximum absorption capacity (mg·g^−1^), *K_L_* and *K_F_* are the Langmuir and Freundlich constants, respectively, and *1/n_F_* is an empirical constant indicating the intensity of adsorption.

Both isotherms curves for crystal violet adsorption are presented in the Supplementary Material, Figure S2.

In order to establish the best equations that describe the adsorption process, values of determination coefficient (R^2^), sum of square error (SSE), chi-square (χ^2^) and average relative error (ARE) were considered [57]. The criterion for their applicability was the higher value for *R^2^* and the lower values for *SSE*, *χ^2^*, and *ARE*. These parameters were determined with equations (5)−(8) [57].

(5) $$R^{2}=1-\frac{\sum_{i=1}^{n}{\left(y_{i,exp}-y_{i,mod}\right)}^{2}}{\sum_{i=1}^{n}{\left(y_{i,exp}-\bar{y_{i,exp}}\right)}^{2}},$$

(6) $$SSE=\sum_{i=1}^{n}{\left(y_{i,exp}-y_{i,mod}\right)}^{2},$$

(7) $$\chi^{2}=\sum_{i=1}^{n}\frac{{\left(y_{i,exp}-y_{i,mod}\right)}^{2}}{y_{i,mod}},$$

(8) $$ARE=\frac{100}{n}\sum_{i=1}^{n}\left|\frac{y_{i,exp}-y_{i,mod}}{y_{i,mod}}\right|,$$

where: *y_i,exp_* is the experimental value, *y_i,mod_* is the modeled value, $\bar{y_{i,exp}}$ is the mean values, and *n* is the total amount of information.

The values of (R^2^), (SSE), (χ^2^) and (ARE) are summarized in Table 3. It can be concluded that Langmuir isotherm best describes the adsorption process. 

The *R_L_* separation coefficient can be used to predict whether an adsorption system is favorable or unfavorable. It can be calculated using the following equation:

(9) $$R_{L}=\frac{1}{1+K_{L}\cdot C_{0}},$$

where: *K_L_* is the Langmuir constant, and C_0_ is the initial dye concentration (mg·L^−1^) [10,11,22].

The calculated value for *R_L_* (0.29) indicates a favorable adsorption.

The value of the maximum adsorption capacity obtained for Bathurst burr powder, 164.10 (mg·g^−1^), is higher than other similar adsorbents previously reported in the literature. Table 4 shows the values for the maximum adsorption capacities of several similar adsorbents used for the adsorption of crystal violet dye.

### 3.5. Kinetic and Thermodynamic Studies

The adsorption kinetics study provides information on the operating conditions and the kinetic parameters. These are the basis for predicting the adsorption rate and designing the adsorption processes [52,59].

**Figure 5:**
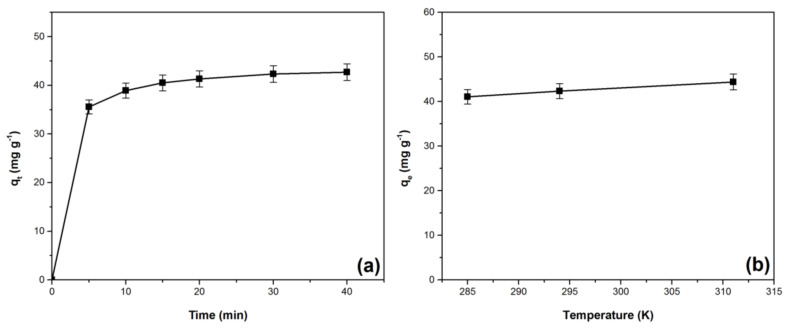
Effect of (**a**) contact time and (**b**) temperature on adsorption capacity for the crystal violet adsorption onto Bathurst burr powder [adsorption conditions: (**a**): pH 7; initial dye concentration: 100 mg·L^−1^; adsorbent dose: 2 g·L^−1^; temperature: 294 K, ionic strength: 0 mol L^−1^ (**b**): pH 7, initial dye concentration: 100 mg·L^−1^; contact time: 30 min; adsorbent dose: 2 g·L^−1^; ionic strength: 0 mol L^−1^].

The effect of contact time on crystal violet adsorption is shown in Figure 5a. The adsorption capacity increases with the contact time between the dye solution and the adsorbent material. After 30 min, the equilibrium is reached and the value of this parameter remains practically constant. In the first 15 min the increase is more pronounced because at the beginning of the process many free adsorption sites on the surface of the adsorbent are available to retain the dye, [8,9,24,42,49]. After 15 min the adsorption capacity increases more slowly, because more and more free sites on the adsorbent surface are occupied by dye molecules and repulsive forces between adsorbed and liquid phase molecules may occur [8,9,45,50]. The increase of the adsorption capacity stops when the equilibrium is reached, the whole adsorbent surface becomes saturated with dye, and the adsorption sites are all occupied [8,9,47].

The equilibrium times, reported in scientific literature, for different crystal violet adsorbents, were: 15 min for pecan pericarp [3]; 30 min for *Artocarpus odoratissimus* leaf-based cellulose [43] and graphene oxide intercalated montmorillonite nanocomposite [60]; 45 min for functionalized multi-walled carbon nanotubes [50]; 60 min for *Moringa oleifera* pod husk [24]; 90 min for NaOH-modified rice husk [45]; 120 min *for Ocotea puberula* bark powder [5], *Terminalia arjuna* sawdust [9], and pinus bark powder [14]; 150 min for yeast-treated peat [44]; 180 min for *Eragrostis plana* nees [7]; 240 min for lemongrass leaf combined with cellulose acetate [42]; and 420 min for crosslinked grafted xanthan gum [48].

The pseudo-first-order and pseudo-second-order models, in the form of non-linear equations [7,8,43,55,56,57] were employed to study the adsorption kinetics:

(10) $$\mathrm{Pseudo}-\mathrm{first}-\mathrm{order}\mathrm{model}\mathrm{equation}:q_{t}=q_{e}(1-{exp}^{-k_{1}\cdot t}),$$

(11) $$\mathrm{Pseudo}-\mathrm{second}-\mathrm{order}\mathrm{model}\mathrm{equation}:q_{t}=\frac{k_{2}\cdot t\cdot q_{e}^{2}}{1+k_{2}\cdot t\cdot q_{e}},$$

where: *q_t_* is the crystal violet amount adsorbed at time *t* (mg·g^−1^), and *k_1_* and *k_2_* are the rate constants of pseudo-first-order kinetic model and the pseudo-second-order kinetic model, respectively.

Both kinetic model curves for crystal violet adsorption are presented in the Supplementary Material, Figure S3.

The pseudo-second-order kinetic model had the highest value for the determination coefficient (R^2^) and the lowest values for sum of square error (SSE), chi-square (χ^2^), and average relative error (ARE), (Table 5). Therefore, this model is the most suitable to characterize the process. A similar observation has been reported in other previous scientific articles [7,11,14,16,18,19,25].

Figure 5b illustrates the effect of temperature on the adsorption capacity, indicating that the adsorption process is endothermic in nature [61,62]. A similar aspect has been reported in other previous studies regarding crystal violet absorption [44,54]. The increase of the adsorption capacity can be explained based on the increasing mobility of the dye molecules due to the viscosity decrease with temperature [44,47,54,62].

Standard Gibbs free energy change, standard enthalpy change, and standard entropy change were calculated from experimental data obtained at temperatures of 285, 294, and 311 K using the equations described in literature [7,56]:

(12) $$\Delta G^{0}=-RTlnK_{L},$$

(13) $$lnK_{L}=\frac{\Delta S^{0}}{R}-\frac{\Delta H^{0}}{RT},$$

where: *R* is the universal gas constant, *K_L_* is the Langmuir constant, and *T* is the absolute temperature.

Thermodynamic parameters (Table 6) were determined from the slope and the intercept of ln K_L_ versus 1/T plot (see Supplementary Material, Figure S4). The data analysis show that the adsorption process is endothermic, spontaneous, and favorable (ΔH^0^ > 0, ΔG^0^ < 0). The affinity of adsorbent for crystal violet and the increased randomness at the solid–solute interface (the degrees of freedom of the adsorbed species) are indicated by the positive value of ΔS^0^ [1,25,56]. Similar results were obtained by other researchers on low-cost adsorbent materials [7,14,25]. The physisorption is involved in the process (ΔH^0^ < 40 kJ mol^−1^) [25,44] and van der Waals interaction plays an important role in the physical adsorption (ΔH^0^ < 20 kJ mol^−1^) [63]. When standard Gibbs free energy change (ΔG^0^) ranges from −20 to −80 (kJ mol^−1^) the process is physisorption, intensified by the chemical effect [49].

### 3.6. Optimization Parameters of Adsorption Process Using Taguchi Approach

The Taguchi method was used to determine the optimal experimental conditions for obtaining the highest efficiency of dye removal from water, using a minimized number of experiments. According to this method, the experimental results are converted into a signal-to-noise (S/N) ratio, which is an indicator that describes at the same time the level of dispersion and degree of optimization, in relation to the desired value. Depending on the number of controllable parameters and their levels, the Taguchi method uses the proper and most representative orthogonal array that distributes the variables in a balanced way [30,31,32]. For six controllable factors at three levels, the classical full factorial design must use more experiments (3^6^ = 729) to establish optimal conditions. In this case, the proper Taguchi orthogonal array is L_27,_ which reduce the number of experiments to 27.

**Figure 6:**
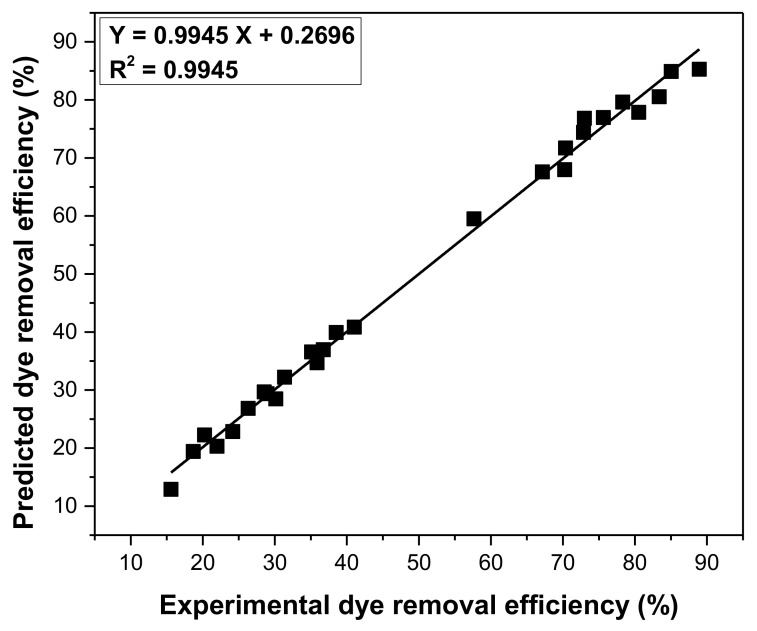
Comparison of experimental and predicted dye removal efficiency.

The obtained results for dye removal efficiency and the S/N ratios for each run are presented in Supplementary Material, Table S1. The S/N ratio rank was used to establish the order of the controllable factors’ significance (Table 7). The factor that had the least influence on the process was the temperature, while the factor with the greatest influence was the pH. The optimal conditions to obtain the highest efficiency of crystal violet removal are highlighted in Table 7. ANOVA results for signal-to-noise S/N ratios certify the same order of influence for the controllable factor. The percentage contribution of each factor is shown in Table 7. The predicted crystal violet removal efficiency was correlated with the experimentally determined one, and it was found that the accuracy of the prediction of the Taguchi method is very good, R^2^ being close to unity (Figure 6).

### 3.7. Desorption Study 

The regeneration possibility of the used adsorbent was performed using three desorbing agents (0.1 M HCl, distilled water, and 0.1 M NaOH). HCl was found as the best desorption agent (see Supplementary Information, Figure S5) but the value of desorption efficiency was lower than 40%. Based on these results and taking into account that Bathurst burr powder is a cheap and easily available material and that desorption involves costs associated with the desorption reagents, it can be concluded that regeneration is not recommended.

The saturated adsorbent, based on its combustion properties as a vegetable material, can be used for direct combustion in specialized incinerators. Another new alternative is to use it as foaming agent for porous glass and ceramic materials based on the gases that result during the thermal synthesis process.

## 4. Conclusions

The natural adsorbent material, Bathurst burr powder, can be successfully used for crystal violet dye removal from aqueous solution. The removal efficiency and the adsorption capacity of this material are influenced by pH, contact time, initial dye concentration, adsorbent dose, and ionic strength. The adsorption process was found to follow Langmuir isotherm and pseudo-second order kinetic model. Maximum adsorption capacity value was higher compared to many similar adsorbents reported in the literature. The adsorption process is endothermic, spontaneous, and favorable. A physisorption mechanism, intensified by the chemical effect, is involved, and the van der Waals interaction plays an important role in the physical adsorption. The Taguchi method shows that the most influential controllable factor was the pH, while the least influential controllable factor was the initial dye concentration. The correlation between the predicted and the experimentally determined dye removal efficiency indicates a very good accuracy of the Taguchi experimental design.

## Figures and Tables

**Table 1:** Controllable factors and their levels.

Factor	Level 1	Level 2	Level 3
pH	2	6	12
Time (min)	5	20	40
Adsorbent dose (mg·L^−1^)	0.5	2.0	3.0
Initial dye concentration (mg·L^−1^)	50	150	250
Temperature (K)	285	294	311
Ionic strength (mol L^−1^)	0	0.10	0.25

**Table 2:** The FTIR bands that were assigned to different cellulose and hemicellulose.

FTIR Bands	Assignment	Reference
3448 cm^−1^	O–H stretching	[31,36,38]
2340 cm^−1^	O–H bending of adsorbed water	[32]
1630 cm^−1^	O–H bending of adsorbed water	[33]
1368 cm^−1^	C–H bending	[34,36,37]
1000 cm^−1^	C–O stretching	[35]

**Table 3:** Adsorption isotherms model constants and the corresponding error functions.

Isotherm Model	Parameters	Value
**Langmuir non-linear**	K_L_ (L mg^−1^)	0.024 ± 0.001
	q_max_ (mg·g^−1^)	164.1 ± 4.23
	R^2^	0.9967
	χ^2^	0.222
	SSE	11.47
	ARE (%)	2.57
**Freundlich non-linear**	K_f_ (mg·g^−1^)	8.62 ± 1.47
	1/n	0.60 ± 0.03
	R^2^	0.9828
	χ^2^	1.249
	SSE	62.12
	ARE (%)	6.94

**Table 4:** Maximum adsorption capacities values of similar adsorbents used for the crystal violet dye adsorption.

Adsorbent	Maximum Adsorption Capacity (mg·g^−1^)	Reference
Water hyacinth root powder	322.58	[20]
Used black tea leaves	200.00	[58]
**Bathurst burr powder**	**164.10**	**This study**
*Moringa oleifera* pod husk	156.25	[24]
Breadfruit skin	145.80	[8]
Papaya seeds	85.99	[19]
Pará chestnut husk	83.60	[21]
Pineapple leaf powder	78.22	[18]
Wheat bran	69.15	[13]
*Laminaria japonica*	66.64	[13]
Coir pith	65.53	[15]
*Eragrostis plana* nees	60.10	[7]
Jackfruit leaf powder	43.39	[17]
Rice bran	41.68	[13]
Sawdust	37.83	[15]
Date palm leaves powder	37.73	[22]
Peel of *Cucumis sativa* fruit	34.24	[16]
Coniferous pinus bark powder	32.78	[14]
Cedar cones	13.64	[11]
Almond shells	12.20	[25]
Sugarcane fiber	10.44	[15]
Corn stalk	9.64	[23]
*Calotropis procera* leaf	4.14	[12]

**Table 5:** Kinetic model constants and the corresponding error functions.

Kinetic Model	Parameters	Value
**Pseudo-first order non-linear**	k_1_ (min^−1^)	0.371 ± 0.021
	q_e,calc_ (mg·g^−1^)	41.40 ± 0.62
	R^2^	0.9960
	χ^2^	0.138
	SSE	5.56
	ARE (%)	16.09
**Pseudo-second order non-linear**	k_2_ (min^−1^)	0.019 ± 0.008
	q_e,calc_ (mg·g^−1^)	43.85 ± 0.21
	R^2^	0.9999
	χ^2^	0.007
	SSE	0.14
	ARE (%)	14.59

**Table 6:** Thermodynamic parameters for the crystal violet adsorption onto Bathurst burr powder.

ΔG (kJ·mol^−1^)			ΔH (kJ·mol^−1^)	ΔS (J mol^−1^·K^−1^)
285 K	294 K	311 K		
−21.62	−22.51	−24.35	1.01	12.66

**Table 7:** Response table for signal-to-noise S/N ratios (larger is better) and contribution percentage of controllable factor influence on crystal violet removal process.

Level	pH	Ionic Strength	Adsorbent Dose	Initial Dye Concentration	Time	Temperature
1	27.40	33.76 *	29.14	32.97 *	31.75	32.40
2	35.04	32.36	34.34	32.96	33.05	32.66
3	35.70 *	32.01	34.65 *	32.19	33.33 *	33.07 *
Delta	8.30	1.74	5.50	0.78	1.59	0.68
Rank	1	3	2	5	4	6
Contribution (%)	65.00	2.60	29.23	0.62	2.19	0.36

* The maximum mean S/N ratio indicative of optimum condition.

## Data Availability

All the experimental data obtained are presented, in the form of table and/or figure, in the article and in the Supplementary materials.

## Supplementary Materials

The following are available online at https://www.mdpi.com/article/10.3390/ma14195861/s1, Table S1: Experimental layout of L27 orthogonal array and results obtained for removal efficiency and S/N ratios, Figure S1: The particle size distribution of the Bathurst burr powder, Figure S2: Langmuir and Freundlich adsorption isotherms (non-linear forms) for the crystal violet adsorption onto Bathurst burr powder, Figure S3: Pseudo-first-order and pseudo-second-order kinetic models (non-linear forms) for the crystal violet adsorption onto Bathurst burr powder, Figure S4: Plot of ln K_L_ vs. 1/T for the crystal violet adsorption onto Bathurst burr powder, Figure S5: The desorption efficiencies of crystal violet dye in different media.
