# Supplementary material for: Bathurst Burr (Xanthium spinosum) Powder—A New Natural Effective Adsorbent for Crystal Violet Dye Removal from Synthetic Wastewaters
Source: Materials (Basel). 2021 Oct 7;14(19):5861. doi: 10.3390/ma14195861 (PMC8510156; doi:10.3390/ma14195861)
Supplement: Supplementary file 1 [file materials-14-05861-s001.zip › materials-1372039-supplementary.pdf]

materials

Article

Bathurst Burr (*Xanthium Spinosum*) Powder—a New Natural Effective Adsorbent for Crystal Violet Dye Removal from Synthetic Wastewaters

Giannin Mosoarca [1], Cosmin Vancea [1,*], Simona Popa [1,*] and Sorina Boran [1]

Faculty of Industrial Chemistry and Environmental Engineering, Politehnica University Timisoara, Bd. V. Parvan, No. 6, 300223, Timisoara, Romania; giannin.mosoarca@upt.ro (G.M.); sorina.boran@upt.ro (S.B.)

* Correspondence: cosmin.vancea@upt.ro, Tel.: +40256404194 (C.V.); simona.popa@upt.ro, Tel.: +40256404212 (S.P.)

Citation: Mosoarca, G.; Vancea, C.; Popa, S.; Boran, S. Bathurst Burr (*Xanthium Spinosum*) Powder a New Natural Effective Adsorbent for Crystal Violet Dye Removal from Synthetic Wastewaters. *Materials* **2021**, *14*, x. https://doi.org/10.3390/xxxxx

Academic Editor: Dimitrios Papoulis

Received: 23 August 2021
Accepted: 5 October 2021
Published:

Publisher's Note: MDPI stays neutral with regard to jurisdictional claims in published maps and institutional affiliations.

Copyright: © 2021 by the authors. Licensee MDPI, Basel, Switzerland. This article is an open access article distributed under the terms and conditions of the Creative Commons Attribution (CC BY) license (http://creativecommons.org/licenses/by/4.0/).

Table S1. Experimental layout of L27 orthogonal array and results obtained for removal efficiency and S/N ratios.

pH	Ionic Strength	Adsorbent Dose	Initial Dye Concentration	Time	Temperature	Removal Efficiency	S/N Ratio
2	0	0.5	50	5	285	15.6	23.86
2	0	0.5	50	20	304	18.68	25.42
2	0	0.5	50	40	311	20.25	26.12
2	0.1	2	150	5	285	24.16	27.66
2	0.1	2	150	20	304	28.92	29.22
2	0.1	2	150	40	311	31.35	29.92
2	0.25	3	250	5	285	21.98	26.84
2	0.25	3	250	20	304	26.32	28.40
2	0.25	3	250	40	311	28.53	29.10
6	0	3	150	5	304	72.99	37.26
6	0	3	150	20	311	88.95	38.98
6	0	3	150	40	285	85.04	38.59
6	0.1	0.5	250	5	304	30.14	29.58
6	0.1	0.5	250	20	311	36.73	31.30
6	0.1	0.5	250	40	285	35.12	30.91
6	0.25	2	50	5	304	57.65	35.21
6	0.25	2	50	20	311	70.25	36.93
6	0.25	2	50	40	285	67.16	36.54
12	0	2	250	5	311	72.86	37.24
12	0	2	250	20	285	78.3	37.87
12	0	2	250	40	304	83.38	38.42
12	0.1	3	50	5	311	70.37	36.94
12	0.1	3	50	20	285	75.62	37.57
12	0.1	3	50	40	304	80.53	38.11
12	0.25	0.5	150	5	311	35.87	31.09
12	0.25	0.5	150	20	285	38.54	31.71
12	0.25	0.5	150	40	304	41.04	32.26

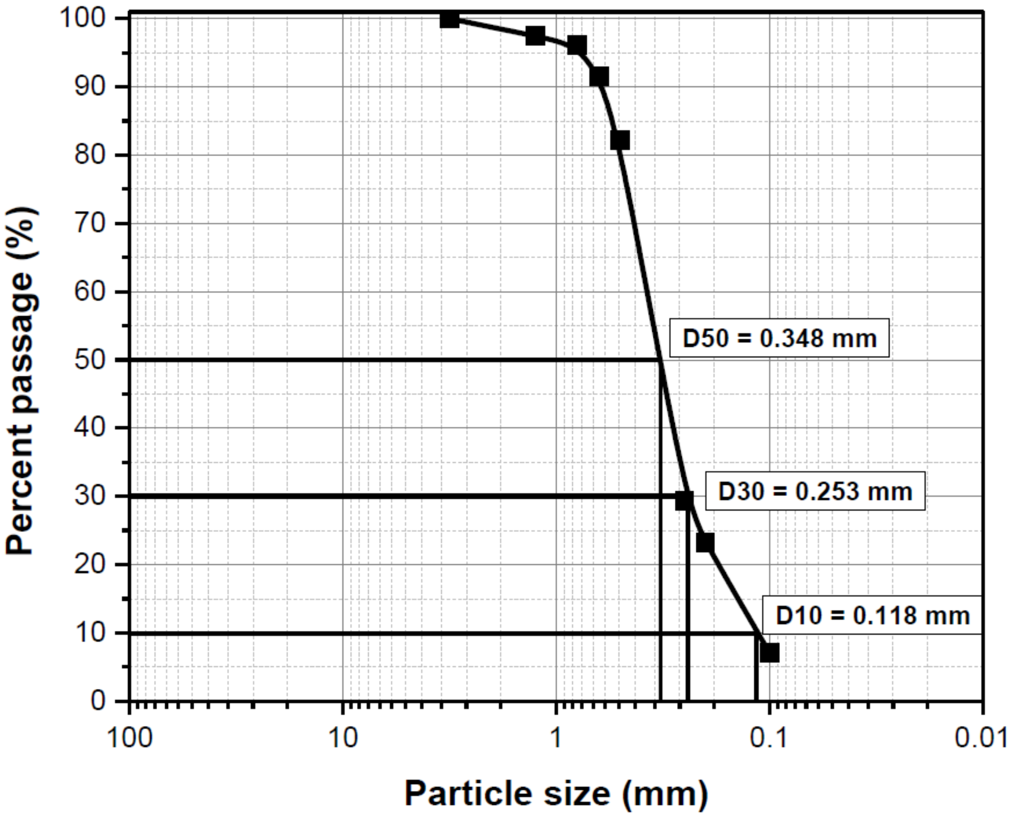

Figure S1. The particle size distribution of the bathurst burr powder.

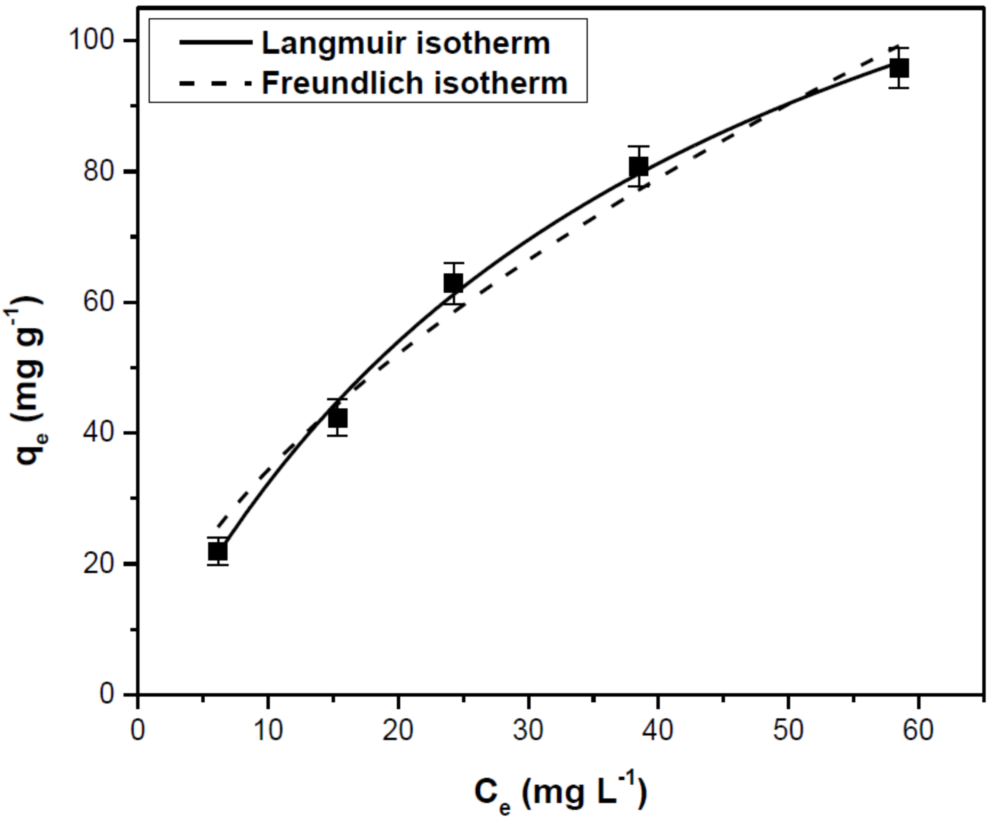

Figure S2. Langmuir and Freundlich adsorption isotherms (non-linear forms) for the crystal violet adsorption onto bathurst burr powder.

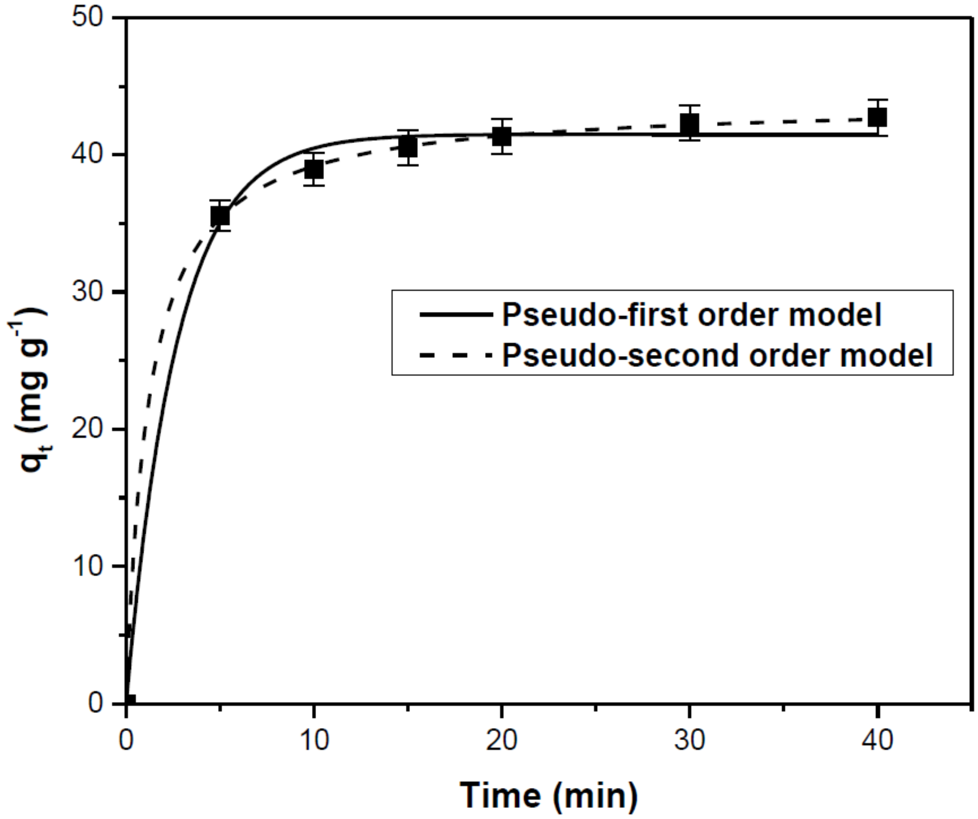

Figure S3. Pseudo-first-order and pseudo-second-order kinetic models (non-linear forms) for the crystal violet adsorption onto bathurst burr powder.

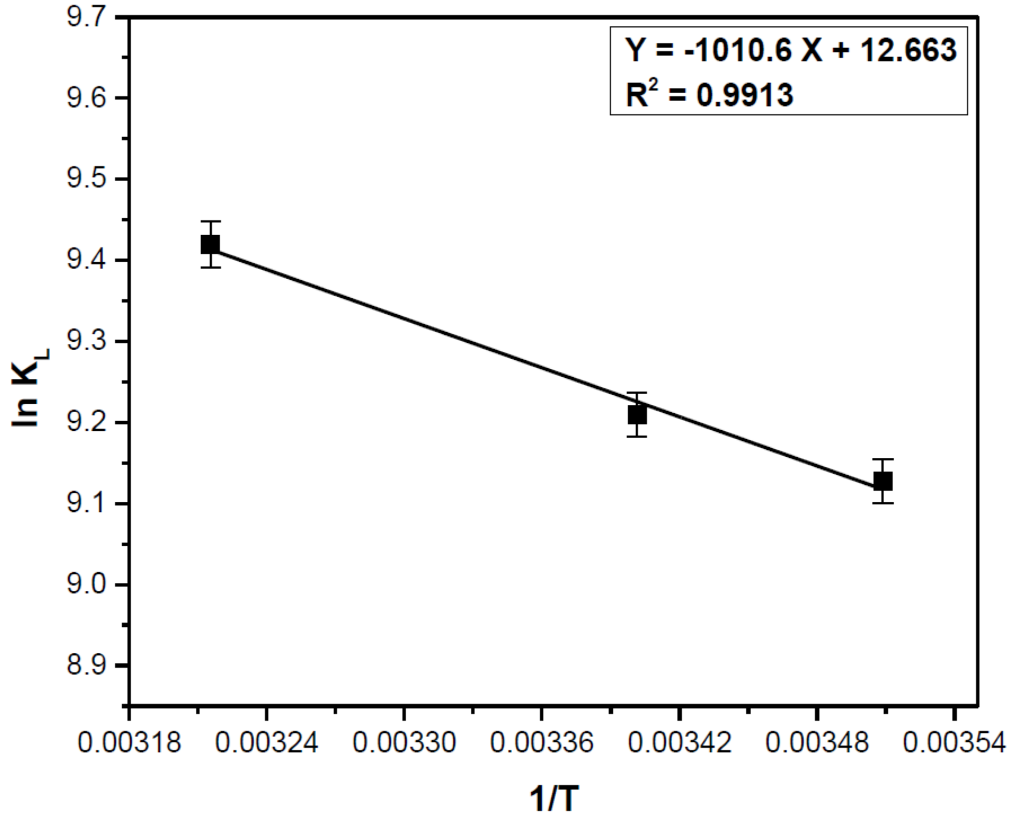

Figure S4. Plot of ln K_L vs. 1/T for the crystal violet adsorption onto bathurst burr powder.

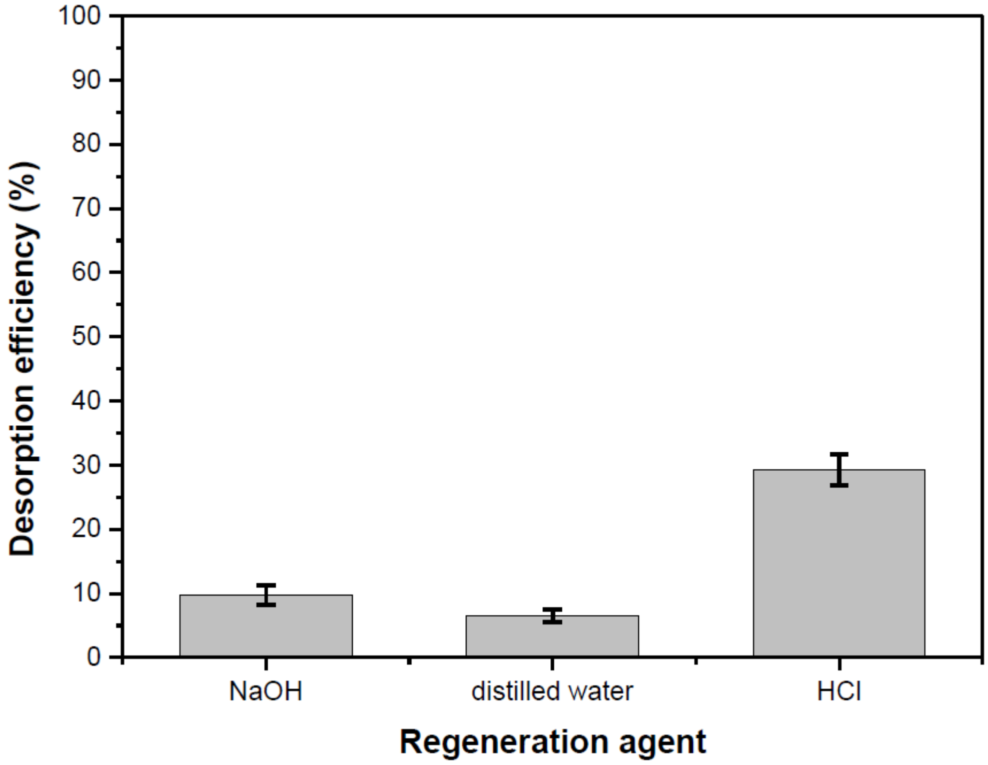

Figure S5. The desorption efficiencies of crystal violet dye in different media.
